# Low-Grade Chondrosarcoma of the Posterior Cricoid Plate

**DOI:** 10.7759/cureus.15400

**Published:** 2021-06-02

**Authors:** Christy M Moen, Alison E Lim, Richard B Townsley

**Affiliations:** 1 Ear, Nose and Throat, University Hospital Crosshouse, Kilmarnock, GBR; 2 Otolaryngology, Queen Elizabeth University Hospital, Glasgow, GBR; 3 Otolaryngology - Head and Neck Surgery, University Hospital Crosshouse, Kilmarnock, GBR

**Keywords:** bone cancer surgery, otolaryngology-head & neck surgeons, total laryngectomy, partial airway obstruction, multi-disciplinary teams

## Abstract

This case report presents an unusual case of chondrosarcoma arising from the cricoid cartilage of the larynx. Although these are commonly low-grade malignancies, this patient attended an outpatient respiratory clinic with acute airway obstruction, and went on to require a total laryngectomy due to the size of their tumour.

## Introduction

Chondrosarcoma is the second most common primary bone malignancy, encompassing 10%-20% of cases [1]. Chondrosarcomas can be found in the head and the neck, and can involve the skull base, sinuses and facial bones. Laryngeal chondrosarcomas (LC) make up approximately 3% of all chondrosarcomas and less than 0.2% of all laryngeal cancers [1,2]. In over 90% of cases, LCs arise from the hyaline cartilage, with the posterior cricoid cartilage being the most common site of origin [1,2]. LCs have a male to female ratio of approximately 3:1, and commonly present with hoarseness, dyspnoea and neck lump [2]. The aetiology of LCs is unclear; it may be associated with a disorder of the ossification process that may occur when laryngeal cartilage is put under mechanical stress, from the attachments of the intrinsic and extrinsic laryngeal muscles. 

LCs tend to be low-grade and less aggressive, compared to chondrosarcomas found in other parts of the body [3]. Histologically it can be challenging to distinguish low-grade chondrosarcoma from benign chondromas. Chondrosarcomas have a better prognosis in comparison with other laryngeal cancers. The largest population-based analysis in the current literature was carried out by Dubal et al [3]. They found one-year, five-year and 10-year disease-specific survival for their population of 143 patients with Laryngeal chondrosarcomas to be 96.5%, 88.6% and 84.8%, respectively. This was significantly greater in comparison with other laryngeal tumour survival rates.

## Case presentation

**Figure 1:**
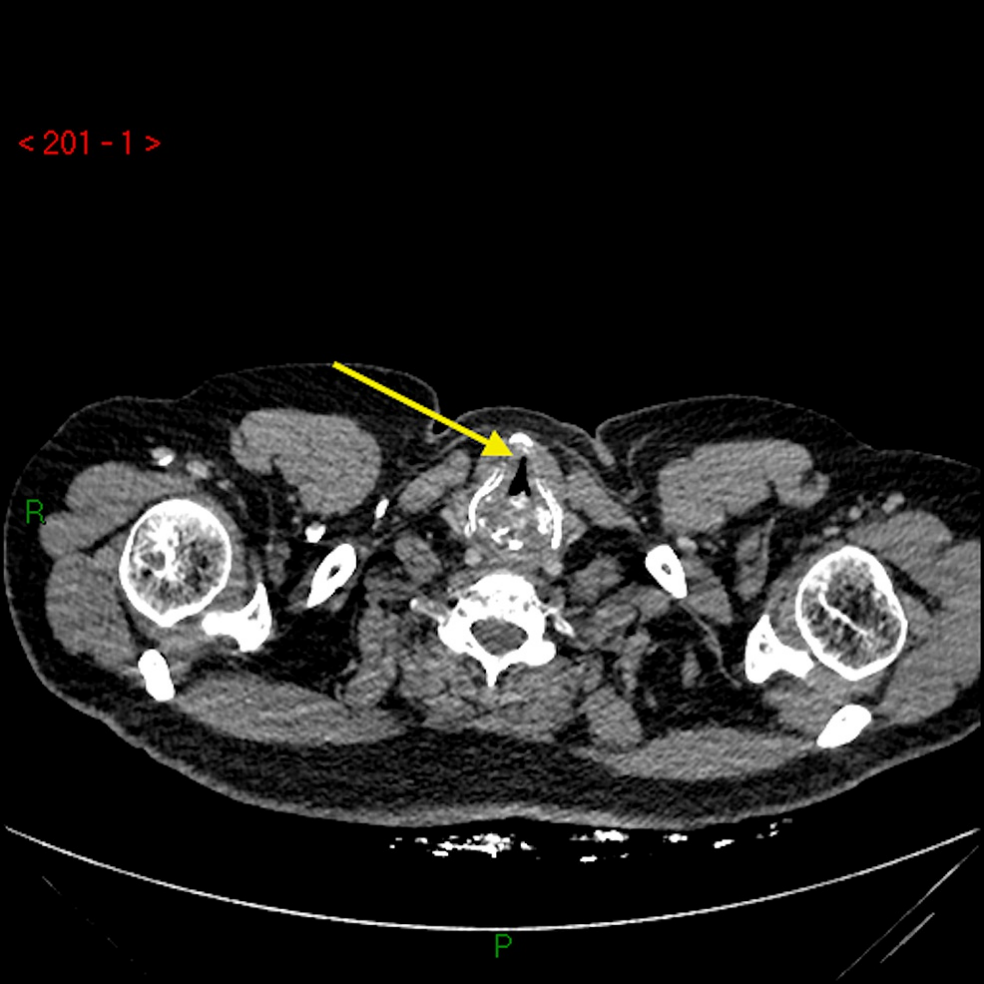
CT demonstrating partially obstructed airway. CT: computed tomography.

**Figure 2:**
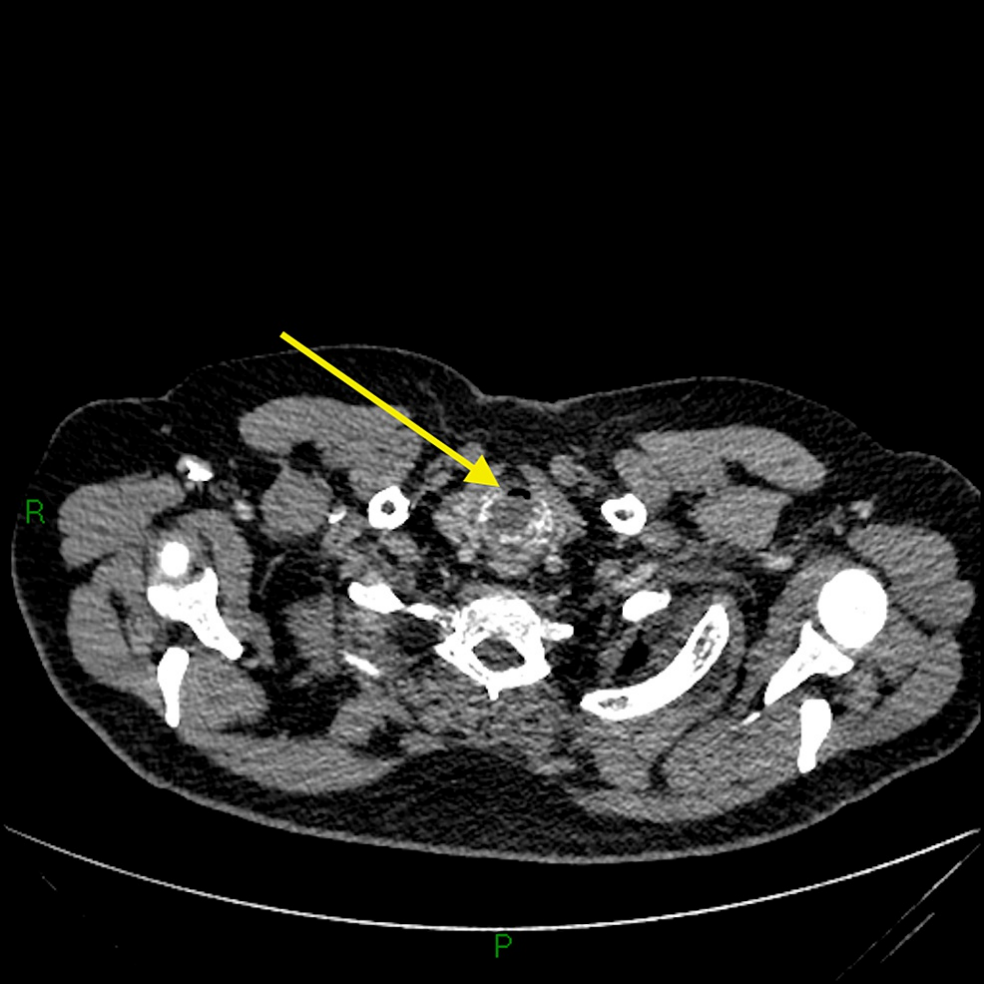
CT demonstrating the minimum diameter of the patient's airway. CT: computed tomography. This CT image demonstrates the minimum luminal airway dimension found which was 8 mm x 3 mm.

Our patient was a 54-year-old gentleman who originally presented with hoarseness and cough to the Otolaryngology clinic where he had no red flag symptoms and no abnormalities seen on flexible nasoendoscopy (FNE). He was reassured and discharged. He re-presented to primary care 8 months later with a persistent cough and shortness of breath and was referred to the respiratory service. On review in the respiratory clinic, he was found to be stridulous with fixed obstructive pulmonary function tests (PFTs), and an urgent computerised tomography (CT) neck, chest and abdomen were arranged. CT scan found a large, 2.8 cm craniocaudal x 3.8 cm transverse x 3 cm anteroposterior, submucosal posterior pharyngeal mass with almost complete occlusion of the subglottic airway, with a minimum luminal airway dimension of 8 mm x 3 mm (Figures 1, 2). 

The patient was admitted immediately and then taken to the emergency theatre due to his critical airway. Awake fibreoptic intubation was attempted, but it was not possible to pass a size 5 micro-endotracheal tube. Local anaesthetic tracheostomy was required to secure the airway before proceeding to general anaesthesia. Biopsies of the large subglottic mass were taken which appeared clinically to be cartilage covered by mucosa. Histopathology results were returned as low-grade chondrosarcoma. He underwent an MRI neck that showed no invasion into local structures or lymph node involvement. 

At this point, the patient was referred to the sarcoma multidisciplinary team meeting (MDT). The conclusion of the MDT was that the patient had a T2N0M0, low-grade chondrosarcoma originating from the posterolateral aspect of the cricoid and that surgery would be offered as a curative treatment option. The patient was admitted electively for a total laryngectomy, primary tracheo-oesophageal puncture and placement of a speech valve. Pathology results confirmed a pT2N0 chondrosarcoma, resected with clear margins. The report also noted small nodules at the base of the epiglottis that may have represented chondromas. The patient recovered well post-operatively and passed a barium swallow test to allow for the removal of his nasogastric tube.

The patient is 18 months post treatment with no signs of recurrence and unrestricted swallowing function.

## Discussion

This is a rare case chondrosarcoma of the cricoid cartilage that was picked up on a CT scan carried out by the respiratory team investigating stridor, demonstrating airway obstruction from a subglottic tumour.

Chondrosarcomas are typically slow-growing, low-grade tumours [4]. In this case, the patient presented with symptoms one year before his airway emergency, with no abnormalities seen on examination at the initial presentation.

Patients with LCs can present with a variety of symptoms, including hoarseness, shortness of breath, dysphagia, and stridor.

A retrospective case study of 111 LCs found that patients present differently depending on the location of the tumour. Patients with endolaryngeal tumours tended to present with shortness of breath, whereas patients with extralaryngeal tumours tended to present with dysphagia [5]. However, Thompson et al found only three of the 111 LCs studied presented with shortness of breath. 

CT scanning is the preferred method of imaging LCs to aid the diagnosis and management planning. Typical LC appearance on a CT scan would show a lesion with ‘popcorn-like’ calcification that enhances with contrast [6].

Surgery is the primary treatment option for chondrosarcomas, and where possible, laryngeal preserving therapies. Tumours of the appropriate size and location can be excised using endoscopic excision or partial laryngectomy [7,8]. If it is not possible to carry out local resection, a total laryngectomy may be required to ensure complete excision of the tumour is achieved.

Of note, Thompson et al [5] found only 1.9% of their patients went on to develop metastases (lung, bone, and liver).

## Conclusions

We have demonstrated an unusual case of chondrosarcoma arising from the cricoid cartilage of the larynx. Although these are commonly low-grade malignancies, this patient presented with acute airway obstruction and required total laryngectomy for a large tumour. Due to the rarity of these cases, it is important to discuss them at regional or national MDTs to ensure the development of experience and expertise of their management.
